# Predictive Role of Fluorescein Angiography in Retinopathy of Prematurity

**DOI:** 10.3390/pediatric16030050

**Published:** 2024-07-24

**Authors:** Gianluca Dini, Alfredo Beccasio, Francesco Della Lena, Alberto Verrotti, Carlo Cagini

**Affiliations:** 1 Department of Pediatrics, University of Perugia, 06129 Perugia, Italy; 2 Department of Medicine and Surgery, Section of Ophthalmology, University of Perugia, S. Maria Della Misericordia Hospital, 06129 Perugia, Italycarlo.cagini@unipg.it (C.C.)

**Keywords:** fluorescein angiography, neonatal care, retinopathy of prematurity (ROP)

## Abstract

Background: Fluorescein angiography (FA) has been a pivotal tool for studying the pathophysiology of retinopathy of prematurity (ROP) in vivo. We examined the course of ROP using FA to assess the predictive value of angiographic features. Methods: This is an observational retrospective cohort study of eyes screened for ROP with a binocular indirect ophthalmoscope and FA. RetCam fundus imaging and video digital fluorescein angiography were performed in the neonatal intensive care unit of Santa Maria Hospital of Perugia. The masked grading of the FA images was retrospectively conducted by two ROP expert ophthalmologists. Results: A total of 80 eyes of 40 patients were included for this study. Among the angiographic features evaluated, leakage, shunts, and tangles were predictive of the development of treatment-requiring ROP (*p* < 0.05). Conclusions: FA can add to our understanding of the evolution of vascular abnormalities in the course of ROP and can help predict which eyes will go on to treatment.

## 1. Introduction

Retinopathy of prematurity (ROP) is a proliferative retinal vascular disease affecting the retina of premature infants [1]. ROP results from a multifactorial impairment of normal retinal development, culminating in the aberrant vascularization of the retina [2,3,4]. The early detection of the retinal vascular pattern in preterm neonates with ROP could play a crucial role in identifying those infants who are likely to require laser treatment [5,6]. Fluorescein angiography (FA) is currently considered a cornerstone in the diagnosis and management of various systemic and ocular diseases involving the retinal vasculature. This imaging modality is based on the intravenous injection of a noniodinated contrast agent followed by the acquisition of serial photographs to visualize the patency and permeability of the retinal vessels. FA was introduced in the 1970s to evaluate ROP thanks to several studies that hypothesized the vascular genesis as the basis of the pathology [7,8,9]. The present study was planned to assess FA as a predictive tool for progression to treatment-severity disease in a group of premature infants.

## 2. Materials and Methods

### 2.1. Study Design

This is a retrospective observational cohort study of premature infants at risk of ROP admitted to the neonatal intensive care unit (NICU) of a tertiary care hospital. All infants born between 2011 and 2022 with a gestational age (GA) ≤ 32 weeks and/or a birthweight (BW) < 1500 g underwent indirect ophthalmoscopy to detect ROP. During this period, all infants with retinal vasculature limited to Zone I and posterior Zone II vascularization underwent FA before 34 weeks postmenstrual age (PMA). Digital fundus images were captured using the RetCam 3 (Clarity Medical Systems Inc., Pleasanton, CA, USA) after achieving pupil dilation with 1 or 2 drops of 0.5% tropicamide administered 15 min before the examination. FA was performed using an intravenous bolus of 10% solution of sodium fluorescein dye (0.1 mL/kg) followed by a saline flush administered via an intravenous cannula under the care of a neonatologist. The procedure and possible risks involved with fundus FA were explained to the parents, and their signed informed consent was obtained.

### 2.2. Grading Parameters

The angiograms were obtained at different time intervals and related to all retinal sectors/quadrants of both eyes. All images were evaluated by two ophthalmologists blinded to BW, GA, and ROP status with only knowledge of fluorescein transit time. Each of the ophthalmologists independently graded the retinal and choroidal lesions of the FA sets, using the criteria reported in a recent work by Lepore [10]. If discordant, a consensus grading was reached by the two graders. The localization and staging of ROP were based on the guidelines outlined in the latest version of ICROP 3 [11].

**Figure 1:**
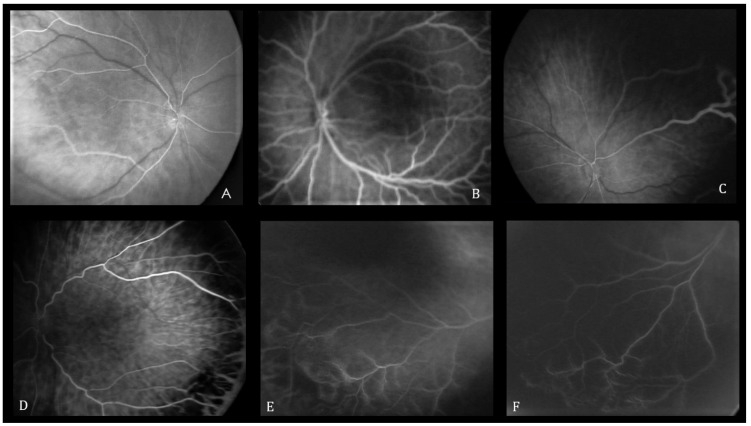
Fluorescein angiography abnormalities within vascularized retina. (**A**) Absence of foveal avascular zone, (**B**) presence of foveal avascular zone, (**C**) linear pattern of choroidal vascular circulation, (**D**) lobular pattern of choroidal vascular circulation, (**E**) hypofluorescent area surrounded by hyperfluorescence, (**F**) hypofluorescent area without surrounding hyperfluorescence.

**Figure 2:**
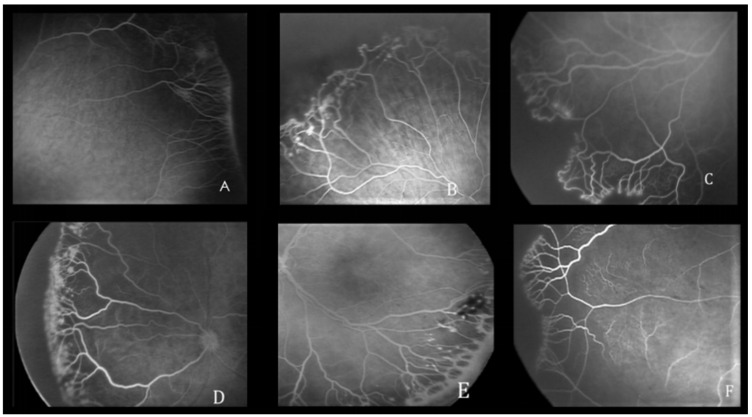
Fluorescein angiography abnormalities at the junction between the vascular and avascular retina. (**A**) Finger-like vascular branching pattern, (**B**) naked arteriovenous shunt, (**C**) capillary tangles, (**D**) hyperfluorescent lesions, (**E**) perivascular dye leakage, (**F**) capillary obliteration.

The features of the vascularized retina are shown in Figure 1 and include the absence or presence of the foveal avascular zone (FAZ), the linear or lobular pattern of choroidal contrast enhancement, and hypofluorescent areas with adjacent hyperfluorescent or hypofluorescent lesions. The FA features of the retinal vascular–avascular junction include finger-like anastomoses, arteriovenous shunts, vascular tangles, leakage, capillary obliteration, and hyperfluorescent lesions (i.e., popcorn lesions and cotton-wool patches) (Figure 2). Patients were treated with laser therapy between 24 and 72 h after the ophthalmoscopic identification of treatment-requiring ROP by two ophthalmologists.

### 2.3. Statistical Analysis

Demographic characteristics such as BW and GA were expressed as the mean (standard deviation) and median. To determine whether FA features were predictive of disease requiring treatment, we analyzed each feature using Fisher’s exact test. *p* < 0.05 was considered statistically significant. For each FA feature, the odds ratio (OR) and its 95% confidence intervals (95% CIs) were calculated. Data analysis was performed using SPSS statistical software version 29.0 (IBM SPSS Statistics).

## 3. Results

### 3.1. Demographic Data

From 1 April 2011 to 31 December 2022, 40 infants were included in this study. The mean GA was 27.1 ± 2.3 weeks (median: 27, range: 23–32), and the mean BW was 952 ± 343.9 g (median: 894, range: 500–2820) (Table 1).

### 3.2. FA Features

Among the 80 eyes examined in this study, finger-like anastomoses were identified in 96.25% (n = 77) and were classified as mild, moderate, or severe depending on the location and density of the vessels [12] (Table 2).

Arteriovenous shunts were identified in 93.75% (n = 75) of preterm infants’ eyes, with severe cases exhibiting shunts connecting all four quadrants of the retina, indicative of severe retinal circulation dysfunction. Capillary tangles, observed in 76.25% (n = 61), were frequently associated with varying levels of leakage. Perivascular dye leakage was identified in 45% (n = 36) of the eyes, categorized as moderate/severe in 17.5% and mild in 27.5%. Regarding the FAZ, it was present in 53.75% (n = 43), absent in 28.75% (n = 23), and not evaluable in 17.5% (n = 14) of the examinations.

Analyzing the association between the presence of FA features and treatment-requiring ROP using Fisher’s exact test revealed several significant findings at the junction of the vascular and avascular retina. The strongest associations were observed with leakage (*p* = 0.008), shunts (*p* = 0.009), and the presence of capillary tangles (*p* = 0.015). Regarding the features of the vascularized portion of the retina, only a borderline association was evident for the hypofluorescent area surrounded by hyperfluorescence (*p* = 0.07).

No adverse events related to the FA contrastographic examination were reported, except for some cases of transient apnea due to pulmonary comorbidity in some newborns or bradycardia triggered by the oculo-vagal reflex from the application of the device’s probe. These issues were promptly resolved with a momentary interruption of the examination.

## 4. Discussion

ROP is a complex condition characterized by abnormal blood vessel growth in the retina of premature infants. Traditionally, diagnosis relied on clinical examination using indirect ophthalmoscopy, but the advent of digital wide-angle imaging systems has transformed ROP evaluation [13].

To ensure that all infants who would benefit from treatment are identified, repeated dilated eye examinations are conducted until the retina is fully vascularized. However, these examinations can be very painful for preterm infants, even when performed by skilled ophthalmologists [14,15,16,17]. With existing criteria, only about 5–10% of screened infants will require treatment [18]. Therefore, efforts to safely reduce the number of stressful and costly screening examinations would be beneficial.

The WINROP algorithm, developed by Hellström and colleagues, is a valuable tool for identifying preterm infants at high and low risk of developing severe ROP early after birth [19,20]. Studies have shown that WINROP has high sensitivity (98.6%) and negative predictive value (99.7%), indicating its ability to accurately identify infants who are unlikely to develop severe ROP [21]. This suggests that the number of screening examinations can be significantly reduced if WINROP is used in combination with traditional screening methods. Currently, WINROP has been used for over 10,000 infants in neonatal intensive care units across several countries. While it has variable specificity and a poor positive predictive value in some studies [22,23,24], it is still a valuable addition to conventional screening guidelines.

FA can supplement indirect ophthalmoscopy examination for the assessment of ROP. Intravenous fluorescein angiography offers a consistent visualization of the retinal vasculature, with greater sensitivity in diagnosing severity and treatment response in ROP by identifying subtle neovascularization and highlighting ischemia and leakage undetectable on indirect ophthalmoscopy [25]. In addition to FA, there are less invasive (non-contact) imaging methods that provide vessel-specific blood flow data and have potential clinical relevance in the context of ROP [26].

The existing literature on FA in ROP has primarily focused on descriptive analyses, revealing clinically undetectable regions of capillary non-perfusion (CNP) and neovascularization [12]. However, there is a lack of substantial data regarding vascular predictors associated with the progression of ROP. We hypothesize that analyzing the progression of vessel development observed on FA in ROP eyes requiring intervention compared to those not needing intervention could yield valuable insights into vascular predictors of disease advancement. In Table 3, we compare our data with the existing literature regarding lesions predictive of treatment in patients with ROP.

The current limitations of FA in NICU settings, such as its time-consuming nature and limited availability, hinder the widespread application of its findings in clinical care. However, the adoption of FA technology is increasing among pediatric ophthalmologists and retina specialists globally. Since the emergence of new bedside imaging techniques, FA has provided an enhanced visualization of vascular abnormalities that may not be easily discernible on ophthalmoscopic fundus exams [27]. In a study by Ng et al. [28] in 2006, clear retinal vascular patterns were successfully captured as part of ROP screening using angiograms. A recent study by Mansukhani et al. [29] highlighted differences in FA findings between eyes treated with anti-VEGF therapy and those that regressed without treatment. Interestingly, they observed frequent abnormal vascular patterns in both groups.

The present study confirms the high level of reproducibility in interpreting FA images of ROP, as previously demonstrated in earlier investigations [30]. In contrast to other studies assessing ophthalmoscopy images, which have shown variability in diagnosis among experienced ROP ophthalmologists [31,32,33], particularly regarding the plus classification, our study found substantial concordance among ROP specialists. This can be attributed to the shared clinical practice between the two ophthalmologists involved in our study, as well as the ability of FA to provide a clearer distinction of certain arterial and venous characteristics, thereby reducing the risk of subjectivity in interpretation.

In the present study, an exceptionally high proportion of eyes needed treatment at the first examination. This can be explained by the fact that ours is a tertiary care center obtaining referrals for ROP treatment from other hospitals of the region. The limitations of this study include its single-center nature and the relatively small number of eyes available for analysis. Moreover, the FA images were evaluated in consensus, and inter-grader as well as intra-grader variability for the images was not assessed. FA findings need to be addressed by a large randomized controlled multi-institutional blinded prospective trial.

## 5. Conclusions

In the forthcoming years, FA could assume a pivotal role in understanding vascular pathology and guiding the prompt management of ROP. The insights gleaned from angiograms offer details not commonly discernible in basic fundus images obtained via indirect ophthalmoscopy. Furthermore, its utility could extend to guiding potential photocoagulation procedures by precisely delineating the extent of ischemic zones. During the follow-up phase, FA could serve as a valuable tool for monitoring vascular growth and assessing treatment efficacy.

## Figures and Tables

**Table 1:** Demographic and ocular characteristics of study infants.

Characteristics	
Birthweight (g)	
Mean (SD)	952 (343.9)
Median	894
Min, max	500, 2820
Gestational age (weeks)	
Mean (SD)	27.1 (2.3)
Median	27
Min, max	23, 32
Treatment status	
Treated	47 (58.7%)
Not treated	33 (41.2%)

**Table 2:** Analysis between FA features and development of treatment-requiring ROP (N = 80 eyes).

FA Characteristics	N Eyes	Treatment (%)	OR (95% CI)	*p* Value *
Foveal avascular zone				1
Absent	23	14 (60.9%)	1.05 (0.39, 2.83)	
Present	43	24 (55.8%)	0.68 (0.28, 1.69)	
Ungradable	14	9 (64.3%)	1.84 (0.52, 6.48)	
Choroidal vascular pattern				0.48
Linear	39	20 (51.3%)	0.86 (0.37, 1.99)	
Lobular	34	21 (61.8%)	1.24 (0.5, 3.07)	
Ungradable	7	6 (85.7%)	4.68 (0.54, 40.89)	
Hypofluorescent areas WITH Hyperfluorescence				0.07
None	41	20 (48.8%)	0.42 (0.17, 1.06)	
Mild	26	18 (69.2%)	1.94 (0.72, 5.22)	
Moderate	10	7 (70%)	1.75 (0.42, 7.33)	
Severe	3	2 (66.7%)	1.42 (0.12, 16.36)	
Hypofluorescent areas WITHOUT Hyperfluorescence				1
None/mild	39	21 (53.8%)	0.61 (0.25, 1.48)	
Moderate	35	22 (62.8%)	1.48 (0.6, 3.65)	
Severe	6	4 (66.7%)	1.45 (0.25, 8.43)	
Finger-like anastomosis				0.56
None	3	1 (33.3%)	0.34 (0.03, 3.88)	
Mild	24	11 (45.8%)	0.47 (0.18, 1.24)	
Moderate	39	25 (64.1%)	1.54 (0.63, 3.78)	
Severe	14	10 (71.4%)	1.96 (0.56, 6.89)	
Shunts				0.009
None/mild	52	26 (50%)	0.33 (0.12, 0.92)	
Moderate	21	16 (76.2%)	2.89 (0.94, 8.92)	
Severe	7	5 (71.4%)	1.85 (0.34, 10.14)	
Tangles				0.015
None	19	7 (36.8%)	0.24 (0.08, 0.73)	
Mild	28	16 (57.1%)	0.98 (0.39, 2.48)	
Moderate	27	19 (70.4%)	2.29 (0.85, 6.13)	
Severe	6	5 (83.3%)	1.85 (0.2, 16.84)	
Hyperfluorescent lesions				0.38
None	66	37 (56.1%)	0.51 (0.15, 1.79)	
Mild	4	4 (100%)	NA	
Moderate/severe	10	6 (60%)	1.06 (0.27, 4.1)	
Leakage				0.008
None	29	11(37.9%)	0.25 (0.1, 0.67)	
Mild	32	22 (68.7%)	2.02 (0.79, 5.17)	
Moderate/severe	19	14 (73.7%)	2.38 (0.76, 7.42)	
Capillary obliteration				0.48
None	27	15 (55.6%)	0.71 (0.28, 1.8)	
Mild	30	22 (73.3%)	2.98 (1.12, 7.95)	
Moderate/severe	23	10 (43.5%)	0.45 (0.17, 1.2)	

* *p* value calculated using Fisher’s exact test, excluding eyes ungradable for foveal avascular zone and choroidal vascular pattern. Statistical significance at *p* < 0.05.

**Table 3:** Comparison of our results with other literature studies.

Study	Number of Patients	Findings Predictive of Treatment-Requiring ROP
Our study	40 (80 eyes)	Leakage, shunts, and tangles.
Hans et al. [5]	50 (99 eyes)	Delayed retinal arterial perfusion and popcorn lesions.
Lepore et al. [10]	56 (98 eyes)	Leakage, shunts, and hyperfluorescent lesions at the junction between the vascular and avascular zone.

## Data Availability

The data that support the findings of this study are available from the corresponding author upon reasonable request.
